# One-year trajectories of nutritional status in perimenopausal women: a community-based multi-centered prospective study

**DOI:** 10.1186/s12889-024-18405-0

**Published:** 2024-03-28

**Authors:** Shujuan Liao, Li Zhao, Chuanya Huang, Anqi Xiong, Weijun Xiong, Yirong He, Xiao Huang, Victoria Hunter, Biru Luo

**Affiliations:** 1 Department of Nursing, West China Second University Hospital, Sichuan University, No. 20, Section 3, People’s South Road, 610041 Chengdu, Sichuan China; 2 Key Laboratory of Birth Defects and Related Diseases of Women and Children, Sichuan University, Ministry of Education, Chengdu, Sichuan China; 3 Department of Health Policy and Management, West China School of Public Health and West China Fourth Hospital, Sichuan University, Chengdu, Sichuan China; 4 Chengdu Zhongke Zhiyong Information Technology Co., LTD, Chengdu, Sichuan China; 5 Sichuan Daily, Chengdu, Sichuan China; 6 Ya’an Polytechnic College, Ya’an, Sichuan China

**Keywords:** Menopause, Nutrition, Body composition, Longitudinal, Community health

## Abstract

**Background:**

Nutritional status is a modifiable factor associated with perimenopausal women’s health and quality of life. Assessing body composition indicators helps to comprehensively understand nutritional status compared with using body mass index (BMI) only. However, few published studies measured the trends in body composition among perimenopausal women.

**Objectives:**

To assess the one-year trajectory of the nutritional status of perimenopausal women and to explore its influential factors.

**Methods:**

A community-based observational study with 3-wave repeated measurements at 6-month intervals was carried out. The nutritional status indicators include weight, body mass index (BMI), and body composition variables. Bioelectrical impedance analysis was used to assess body composition. Repeated measures ANOVA and Chi-square test were used to calculate the changes in nutritional status and generalized estimating equations were performed to explore their influential factors.

**Results:**

2760 participants completed the study. Increasing trajectories in weight (from 56.05 ± 7.55 to 57.02 ± 7.60), fat mass (from 17.99 ± 4.80 to 20.49 ± 4.90), and waist-hip ratio (from 0.86 ± 0.04 to 0.91 ± 0.15) were found (*P* < 0.001). Decreasing trajectories in skeletal muscle (from 20.30 ± 2.38 to 19.19 ± 2.46), protein level (from 7.39 ± 0.79 to 7.06 ± 0.81), and total body water (from 27.87 ± 2.92 to 27.00 ± 3.01) were found (*P* < 0.001). Being married/unmarried with a partner and without negative life events were associated with higher total body water, skeletal muscle, and protein level, while negatively associated with fat mass and waist-hip ratio. Age was positively associated with fat mass (*P* < 0.001). Participants with junior high school education were prone to increased fat mass (*P* = 0.018) compared with those holding primary school education and below. A per capita monthly income of 1500 to 3000 Yuan was associated with higher total body water, skeletal muscle, and protein level (*P* < 0.001) compared with a per capita monthly income of less than 1500 Yuan.

**Conclusion:**

Worsening nutritional status exists in perimenopausal women, which is characterized by increased weight, fat mass, and waist-hip ratio, and decreased skeletal muscle, total body water, and protein level. For greater efficiency, precision nutritional interventions are needed, and recipients should be classified into different risk levels based on their sociodemographic background.

## Introduction

Perimenopause is also named menopausal transition and refers to the period from the onset of functional deficiency of women’s ovaries till no more than 12 months after the last menstruation [1]. It is the critical life stage transitioning from reproductive to older age. In this stage, nutritional status such as appropriate weight and body composition is crucial not only in maintaining and improving current health but also has long-term influence in the remaining one-third of women’s lives. It has been reported that perimenopausal malnutrition is associated with some adverse health consequences, including central adiposity [2], hypertension [3], other cardiovascular diseases [4], insulin resistance [5], type II diabetes [6], and malignancies such as breast cancer [7]. Moreover, nutritional status is also a modifiable factor associated with both longevity and quality of life [8].

Though of great importance, the nutritional status of perimenopausal women is not optimistic. Physically, menopausal transition is characterized by down-regulated estrogen levels and ovarian dysfunction, which are associated with metabolic syndrome, weight gain, and central fat redistribution [9]. Psychosocially, perimenopause usually coincides with middle age, a life stage with a high possibility of negative life events and changes in social roles associated with retirement. Adverse dietary habits [10] and sedentary lifestyles [11] in this specific life stage could further undermine the vulnerable nutritional status of women in the menopausal transition. Thus, the necessity and importance of assessing and managing nutritional status in perimenopause are stressed in this context.

Body mass index (BMI), calculated as body mass (kg) divided by height squared (m²), is currently the most commonly used indicator of nutritional status. Published literature suggests that nearly 60-70% of menopausal women experience weight gain [12], and an average weight gain of about 0.68 kg per year has been reported [13]. Approximately two-thirds of women aged 40 to 59 years are overweight or obese [14]. As we step into the precision nutrition era, the introduction of body composition showed us a proper way to assess one’s nutritional status more comprehensively in addition to using BMI only. The positive association between menopause and body fat mass [15] and the negative association between skeletal muscle mass [16] have been documented by previous studies. Additionally, a small number of studies explored the nutritional status of perimenopausal women longitudinally, providing us with a more specific profile of the nutritional status of women in this particular life stage. One longitudinal study that explored the variation trends in body composition during perimenopause found that body fat mass increases by 1.7% each year, while lean mass decreases by 0.2% every year during the menopause transition [17].

A recent study revealed that Black women gain more weight and have higher blood pressure in their menopausal onset compared with White women [18], which reflects that nutrition is influenced by a wide range of factors such as dietary behaviors, socioeconomic status, age, health condition, physical activities, and lifestyle. However, the majority of published literature on this topic is from well-developed regions such as the American and European countries, leaving research gaps in China, which has the largest number of perimenopausal women in the world with a unique socio-cultural and economic background. The limited information on the nutritional status of Chinese women in the menopausal transition hinders a better understanding of the nutritional needs of this specific population group and creates obstacles to the management of perimenopausal malnutrition.

Our study adopted a longitudinal design to repeatedly assess the nutritional status of community-dwelling perimenopausal women in Sichuan Province, China, with the purposes of (1) displaying the one-year trajectories of weight, body fat mass, waist-hip ratio, skeletal mass, total body water, and protein in perimenopausal women; (2) exploring the socio-demographic influential factors of nutritional status in this specific population group, thus providing data basis for precision nutrition management.

## Methods

### Study design

This is a multi-centered longitudinal observational study with 3 waves of repeated measurements conducted from January 2021 to January 2022. The baseline assessment was carried out in January 2021 (T1), and the follow-up assessments happened in July 2021 (T2), and January 2022 (T3), respectively with a 6-month interval.

### Study setting

This study was carried out in six sub-districts in Chengdu, Sichuan Province, which is a densely populated and important economic zone located in southwest Mainland China. All six included sub-districts are rich in various local delicacies which are characterized by heavy oil, salt, and spice [19], and all share the same casual lifestyle.

### Study population and sampling

This study was carried out among community-dwelling perimenopausal women. The inclusion criteria were (1) between the ages of 40 and 60, (2) lived in the targeted communities for at least 1 year, (3) with variability in the menstrual cycle for at least 1 year, (4) the last menstruation was within 12 months, (5) with intact uterus and at least one ovary, and (6) volunteered for this study. The exclusion criteria were (1) taking antipsychotics, (2) undergoing hormonal therapy or taking oral contraceptives, (3) being treated for menopausal symptoms, and (4) with a history of significant gynecological conditions, including malignancies. In addition, participants with the following conditions were not followed up on the consideration of sample contamination: (1) hospitalized without completing the 3-wave investigation, (2) being treated for menopausal symptoms during the study, and (3) more than 12 months from the last menstruation at any follow-up.

**Fig. 1:**
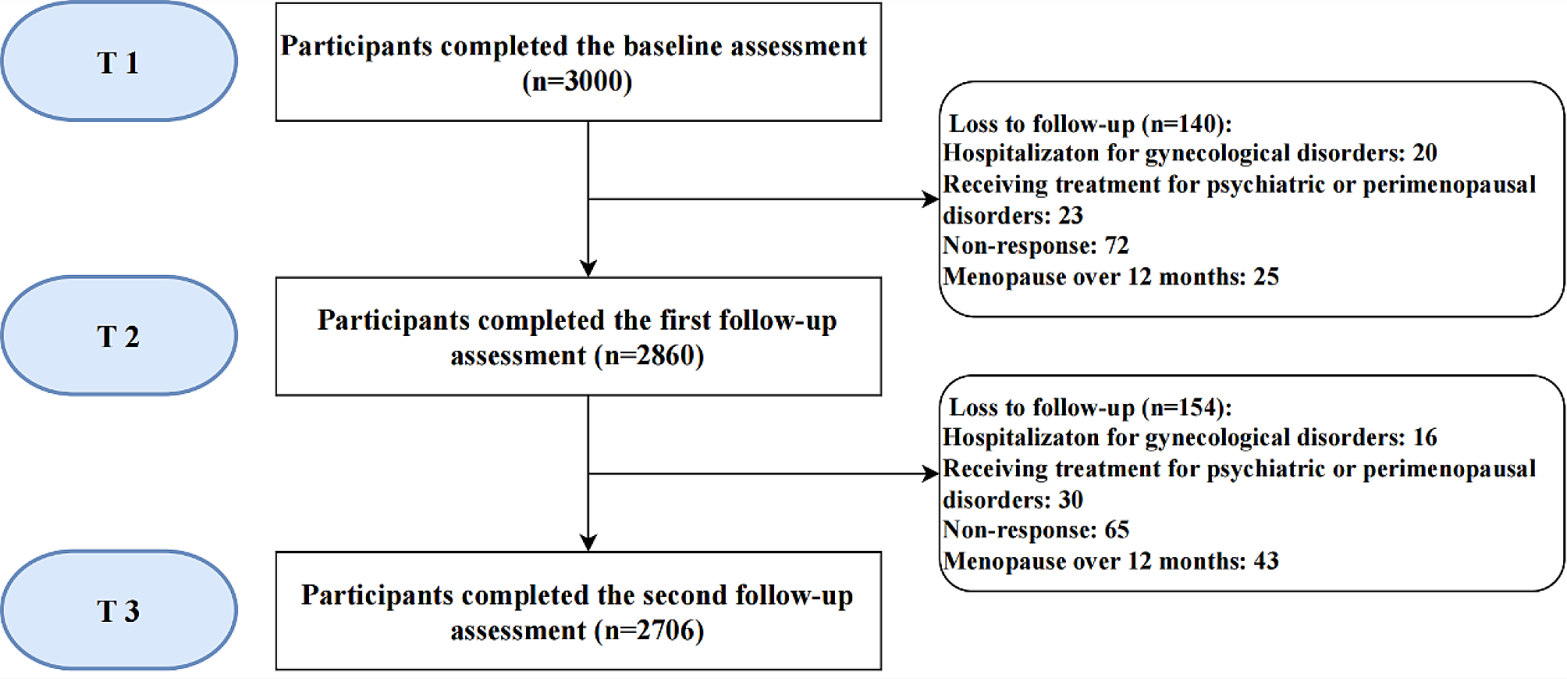
Flowchart of participants

Convenience sampling was adopted in this study. Five hundred qualified women were recruited from each targeted sub-district at T1 (constituting a total sample of 3000); 140 and 154 participants were excluded or lost to follow-up at T2 and T3, respectively (Fig. 1). Finally, 2706 participants completed the entire study with a response rate of 90.2%.

### Measures

#### Sociodemographic data

Sociodemographic information was assessed by a self-developed data collection form, including age (years), education level (classified as primary school and below, junior high school, senior high school or vocational school, and college or above), per capita monthly income (< 1500 Yuan, 1500 to 3000 Yuan, and > 3000 Yuan), marital status (divided into 4 groups: married/unmarried with a partner, divorced, widowed, and single), chronic diseases (yes or no), and negative life events in the past year (yes or no). Chronic diseases are defined as conditions that last for at least one year and require ongoing medical attention and/or limit activities of daily living [20]. Negative life events include the following situations that occurred in the past year: death and/or severe illness of significant others, severe illness of oneself, negative events within relationships, and other loss experiences. Each form requires 1–2 min to complete.

#### Nutritional status

Nutritional status in our study consisted of weight (kg), height (m), BMI, and body composition indicators. For body composition, body fat mass (kg), skeletal muscle mass (kg), total body water (L), protein (kg), and waist-hip ratio were measured.

#### Weight, height, and BMI

Only underwear was allowed while measuring weight and height; shoes were removed. Weight was measured using a digital scale with 0.1 kg of precision. When assessing height, participants were asked to stand straight, heels together, arms hanging down with palms facing thighs, and 0.01 m of precision was adopted. Both weight and height were assessed twice by the same researcher and measuring tools for accuracy. BMI was computed as body mass (kg) divided by height squared (m²). Participants were then classified as underweight (BMI < 18.5 kg/m²), normal weight (18.5 kg/m² ≤ BMI < 24.0 kg/m²), overweight (24.0 kg/m² ≤ BMI < 28.0 kg/m²), or obese (BMI ≥ 28.0 kg/m²) according to the recommendation of the Working Group on Obesity in China [21]. The cutoffs of the Working Group on Obesity in China were chosen as its results were calculated from a Chinese national sample size of 239 972 adults [21]. Due to the absence of samples from Mainland and Taiwan, China, the standard criteria for the Asian population were not used. Assessment of weight and height takes about 1–2 min for each participant.

#### Body composition indicators

Body fat mass, skeletal muscle mass, total body water, protein, and waist-hip ratio were measured using a professional bioelectrical impedance analysis device (InBody230, manufactured in South Korea and imported by Basbex Medical Instrument Trading (Shanghai) Co., LTD). Bioelectrical impedance analysis is a non-invasive, low-cost, and reliable method for body composition assessment in clinical and non-clinical settings [22]. It works based on the rate at which an electrical current travels through one’s body. Specifically, when the electric current encounters fat, it slows due to the greater resistance (impedance) caused by adipose tissue compared with lean mass. Restriction of food and caffeine intake and avoidance of vigorous exercise were required 30 min before testing. When measuring body composition, shoes, socks, and gloves were not allowed. Participants placed each foot on the electrode pad and held the electrode handles in each hand. The time needed for measuring body composition is around 1–2 min.

### Data collection and ethical consideration

Data were collected onsite by trained researchers in the community health centers. The sociodemographic data collection form was completed by each participant independently and returned to the researcher immediately. Researchers explained the exact meaning of each item when questions were raised, and researchers would also read out the items and fill up the form according to participants’ answers for participants with difficulty reading and/or writing. Sociodemographic data were collected only at T1, and nutritional status was repeatedly assessed at T1, T2, and T3. In accordance with the Declaration of Helsinki, our study has been approved by the Medical Ethics Committee of West China Second Hospital of Sichuan University (approval number: 20,220,327). Written informed consent was obtained before recruitment. Participants were fully aware of the purposes of our study and were told they have the right to withdraw at any stage without any negative consequences. Measurement results were available to each participant.

### Statistical analysis

Only those participants with complete data for three waves were included in the data analysis. IBM SPSS version 26.0 was used to perform data analysis. Continuous data were described as Mean with standard deviation (SD). Proportion was used to present categorical data. Repetitive measures ANOVA and Chi-square test were used to compare the differences in nutritional status at T1, T2, and T3. Multivariable linear regressions by generalized estimating equations (GEE) were performed to identify factors associated with nutritional status. A two-tailed *p*-value of less than 0.05 was considered statistically significant.

## Results

### Sociodemographic information of participants

Two thousand seven hundred and six participants completed the study with an average age of 52.42 ± 4.49 years and nearly half of them had a per capita monthly income of less than 1500 Yuan. Most of the participants were at a normal weight (1905 cases, accounting for 70.4%). Our participants were under-educated and their overall educational levels (61.3%) were equal to or lower than junior high school. More than half (51.4%) of them were married or living with a partner, and did not have chronic diseases (54.2%) or negative life events (52.7%). Table 1 presents the sociodemographic information of our participants in detail.

**Table 1:** Sociodemographic information of participants

Variables	Total (*N* = 2706)	Under Weight^1^ (*n* = 258)	Normal weight^2^ (*n* = 1905)	Overweight^3^ (*n* = 471)	Obese^4^ (*n* = 72)	*P*
Age (years)^5^	52.42 ± 4.49	50.67 ± 5.42	52.43 ± 4.44	52.81 ± 4.45	55.82 ± 3.51	< 0.001
Education level^6^						< 0.001
Primary school and below	756 (28.3)	73 (28.3)	531 (27.9)	146 (31.0)	15 (20.8)	
Junior high school	893 (33.0)	74 (28.7)	624 (32.8)	164 (34.8)	31(43.1)	
Senior high or vocational school	46 (16.5)	53 (20.5)	290 (15.2)	83 (17.6)	20 (27.8)	
College or above	602 (22.2)	58 (22.5)	460 (24.1)	78 (16.6)	6 (8.3)	
Per capita monthly income (Yuan)^6^						< 0.001
<1500	1277(47.2)	86(33.3)	922(48.4)	213(45.2)	56(77.8)	
1500 ~ 3000	792(29.3)	77(29.8)	548(28.8)	161(34.2)	6(8.3)	
>3000	637(23.5)	95(36.9)	435(22.8)	97(20.6)	10(13.9)	
Marital status^6^						< 0.001
Married/unmarried with a partner	1392(51.4)	190(73.6)	935(49.1)	215(45.6)	52(72.2)	
Divorced	479(17.7)	40(15.5)	364(19.1)	70(14.9)	5(6.9)	
Widowed	577(21.3)	12(4.7)	465(24.4)	95(20.2)	5(6.9)	
Single	258(9.5)	16(6.2)	141(7.4)	91(19.3)	10(14.0)	
Chronic diseases^6^						0.431
Yes	1238 (45.8)	125 (48.4)	880 (46.2)	201 (42.7)	32 (44.4)	
No	1468 (54.2)	133 (51.6)	1025 (53.8)	270 (57.3)	40 (55.6)	
Negative life events^6^						< 0.001
Yes	1281 (47.3)	119 (46.1)	814 (42.7)	285 (60.5)	63 (87.5)	
No	1425 (52.7)	139 (53.9)	1091 (57.3)	186 (39.5)	9 (12.5)	
Height (m)^5^	1.60 ± 0.05	1.61 ± 0.05	1.60 ± 0.05	1.59 ± 0.05	1.60 ± 0.05	< 0.001
Weight (Kg)^5^	56.05 ± 7.55	45.66 ± 3.31	54.63 ± 4.92	64.56 ± 4.63	75.30 ± 5.94	< 0.001
Total body water (L)^5^	27.87 ± 2.92	24.97 ± 2.12	27.55 ± 2.47	30.04 ± 2.69	32.64 ± 3.03	< 0.001
Skeletal muscle (Kg)^5^	20.30 ± 2.38	17.88 ± 1.71	20.04 ± 2.00	22.10 ± 2.19	24.19 ± 2.45	< 0.001
Fat mass (Kg)^5^	17.99 ± 4.80	11.57 ± 2.00	16.99 ± 3.06	23.56 ± 2.85	30.79 ± 3.56	< 0.001
Protein (Kg)^5^	7.39 ± 0.79	6.59 ± 0.58	7.31 ± 0.66	7.99 ± 0.73	8.69 ± 0.80	< 0.001
Waist-hip ratio^5^	0.86 ± 0.04	0.82 ± 0.03	0.86 ± 0.04	0.89 ± 0.04	0.93 ± 0.04	< 0.001

Note: ^1^BMI < 18.5 kg/m²; ^2^18.5kg/m² ≤ BMI < 24.0 kg/m²; ^3^24.0kg/m² ≤ BMI < 28.0 kg/m²; ^4^BMI ≥ 28.0 kg/m². ^5^analyzed by one way ANOVA. ^6^analyzed by Chi-square test

### One-year trajectories of participants’ nutritional status

**Fig. 2:**
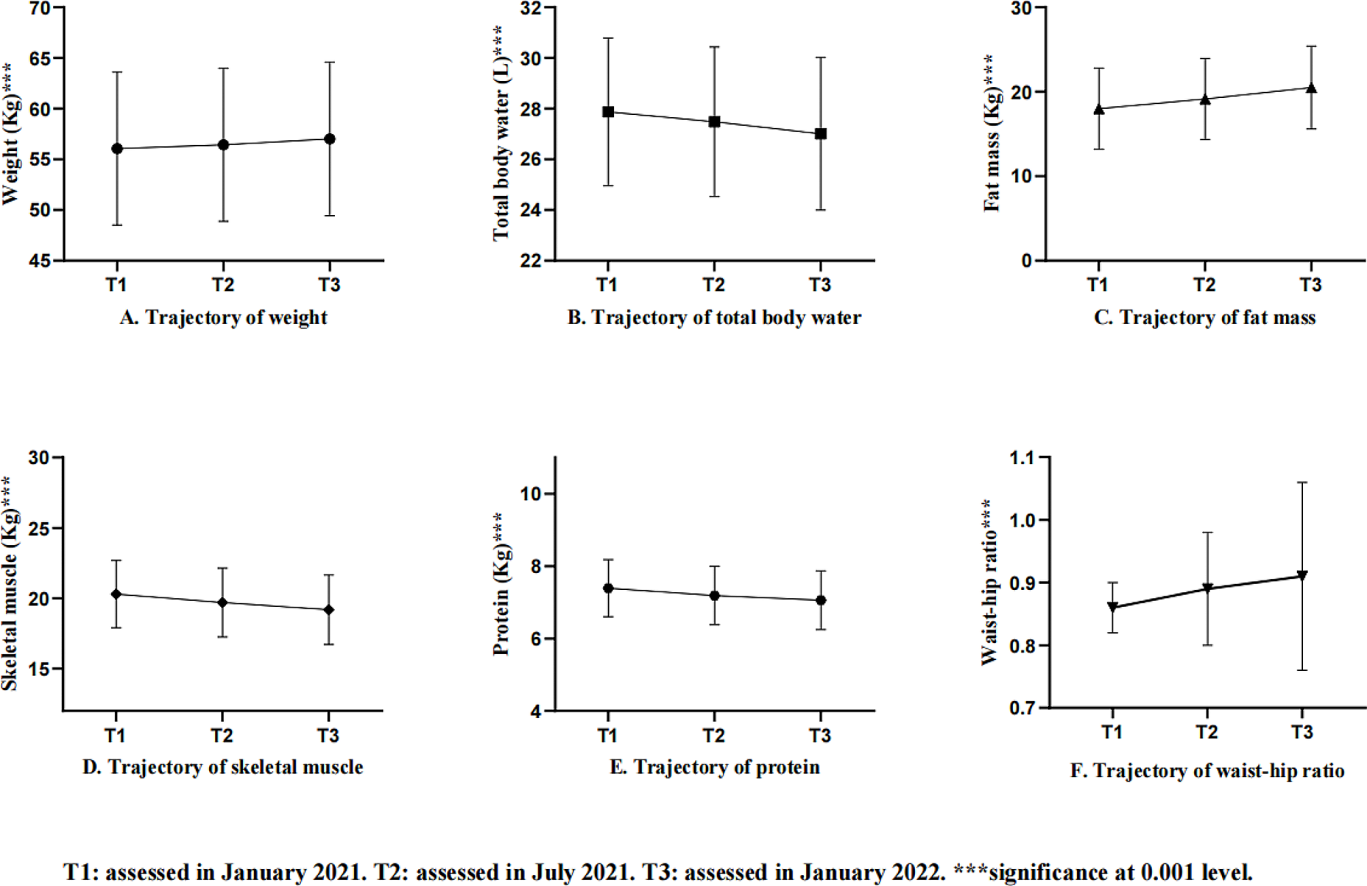
One-year trajectories of participants’ nutritional status

As presented in Table 2, the prevalence of underweight (slightly decreased from 9.5% T1 to 7.7% T3), normal weight (slightly decreased from 70.4% T1 to 69.7% T3), overweight (slightly increased from 17.4% T1 to 19.4% T3), and obese (slightly increased from 2.7% T1 to 3.2% T3) participants did not change statistically throughout the study. Participants’ weight increased from 56.05 ± 7.55 at T1 to 57.02 ± 7.60 at T3 (*P* < 0.001). In terms of specific body composition, the increase in weight was mainly contributed by the accumulation of fat mass (increased from 17.99 ± 4.80 T1 to 20.49 ± 4.90 T3, *P* < 0.001), while downward trajectories were found in total body water (decreased from 27.87 ± 2.92 T1 to 27.00 ± 3.01 T3, *P* < 0.001), skeletal muscle (decreased from 20.30 ± 2.38 T1 to 19.19 ± 2.46 T3, *P* < 0.001), and protein (decreased from 7.39 ± 0.79 T1 to 7.06 ± 0.81 T3, *P* < 0.001). Figure 2 shows in detail the longitudinal trajectories of the participants’ nutritional status.

**Table 2:** Trajectories of participants’ nutritional status (*N* = 2706)

	T1	T2	T3	F/ χ^2^	*p*
BMI^6^				1.586	0.102
Underweight^1^	258 (9.5)	219 (8.1)	208 (7.7)		
Normal weight^2^	1905 (70.4)	1910 (70.6)	1886 (69.7)		
Overweight^3^	471 (17.4)	499 (18.4)	525 (19.4)		
Obese^4^	72 (2.7)	78 (2.9)	87 (3.2)		
Weight (Kg)^5^	56.05 ± 7.55	56.43 ± 7.55	57.02 ± 7.60	3250.756	< 0.001
Total body water (L)^5^	27.87 ± 2.92	27.48 ± 2.95	27.00 ± 3.01	3327.668	< 0.001
Skeletal muscle (Kg)^5^	20.30 ± 2.38	19.70 ± 2.45	19.19 ± 2.46	5868.587	< 0.001
Fat mass (Kg)^5^	17.99 ± 4.80	19.15 ± 4.82	20.49 ± 4.90	10851.932	< 0.001
Protein (Kg)^5^	7.39 ± 0.79	7.19 ± 0.81	7.06 ± 0.81	4596.620	< 0.001
Waist-hip ratio^5^	0.86 ± 0.04	0.89 ± 0.09	0.91 ± 0.15	210.584	< 0.001

Note: ^1^BMI < 18.5 kg/m²; ^2^18.5kg/m² ≤ BMI < 24.0 kg/m²; ^3^24.0kg/m² ≤ BMI < 28.0 kg/m²; ^4^BMI ≥ 28.0 kg/m². ^5^analyzed by repeated measures one way ANOVA. ^6^analyzed by Chi-square test

T1: assessed in January 2021. T2: assessed in July 2021. T3: assessed in January 2022

### Sociodemographic influences on modifiable nutritional status

The influence of sociodemographic data on nutritional status was explored using the generalized estimating equations (GEE) model. All sociodemographic variables gathered from the self-designed form and participants’ height were entered into the model as independent variables, and the modifiable nutritional status indicators (including weight, fat mass, total body water, skeletal muscle, protein level, and waist-hip ratio) were set as dependent variables, respectively. Only those variables significant in each model were presented for concision. As shown in Table 3, height (a relatively stable nutritional variable in perimenopausal women) was positively associated with all other modifiable nutritional indicators in our study (*P* < 0.001). Being married/unmarried with a partner and without negative life events were associated with higher total body water, skeletal muscle, and protein level, while negatively associated with fat mass and waist-hip ratio. Age was positively associated with fat mass (β = 0.11, *P* < 0.001). Participants with junior high school education were prone to increased fat mass (*P* = 0.018) compared with those holding primary school education and below. A per capita monthly income of 1500 to 3000 Yuan was associated with higher total body water, skeletal muscle, and protein level (*P* < 0.001) compared with a per capita monthly income of less than 1500 Yuan.

**Table 3:** Sociodemographic influences on nutritional status (*N* = 2706)

Variables	β	SE	*p*
**Weight**			
Height	58.99	3.30	< 0.001
Per capita monthly income			
>3000	-0.52	0.35	0.140
1500 ~ 3000	1.01	0.34	0.003
<1500	0^a^		
Marital status			
Single	4.56	0.46	< 0.001
Widowed	1.75	0.30	< 0.001
Divorced	0.42	0.34	0.220
Married/unmarried with a partner	0^a^		
Negative life events			
Yes	0.81	0.40	0.042
No	0^a^		
**Total body water**			
Height	30.50	1.32	< 0.001
Per capita monthly income			
>3000	-0.002	0.13	0.988
1500 ~ 3000	0.73	0.12	< 0.001
<1500	0^a^		
Marital status			
Single	-0.20	0.15	0.195
Widowed	-0.46	0.10	< 0.001
Divorced	-0.28	0.12	0.022
Married/unmarried with a partner	0^a^		
Negative life events			
Yes	-0.79	0.13	< 0.001
No	0^a^		
**Skeletal muscle**			
Height	23.74	1.09	< 0.001
Per capita monthly income			
>3000	0.03	0.11	0.765
1500 ~ 3000	0.67	0.10	< 0.001
<1500	0^a^		
Marital status			
Single	-0.08	0.13	0.536
Widowed	-0.33	0.08	< 0.001
Divorced	-0.22	0.10	0.034
Married/unmarried with a partner	0^a^		
Negative life events			
Yes	-0.69	0.11	< 0.001
No	0^a^		
**Protein**			
Height	7.80	0.37	< 0.001
Per capita monthly income			
>3000	-0.02	0.04	0.618
1500 ~ 3000	0.21	0.03	< 0.001
<1500	0^a^		
Marital status			
Single	-0.05	0.04	0.266
Widowed	-0.12	0.03	< 0.001
Divorced	-0.07	0.03	0.052
Married/unmarried with a partner	0^a^		
Negative life events			
Yes	-0.22	0.04	< 0.001
No	0^a^		
**Fat mass**			
Height	17.61	1.98	< 0.001
Age	0.11	0.03	< 0.001
Education level			
College or above	-0.29	0.23	0.198
Senior high or vocational school	0.50	0.28	0.072
Junior high school	0.51	0.21	0.018
Primary school and below	0^a^		
Per capita monthly income			
>3000	-0.53	0.21	0.013
1500 ~ 3000	-0.001	0.21	0.997
<1500	0^a^		
Marital status			
Single	4.86	0.30	< 0.001
Widowed	2.39	0.20	< 0.001
Divorced	0.79	0.21	< 0.001
Married/unmarried with a partner	0^a^		
Negative life events			
Yes	1.88	0.25	< 0.001
No	0^a^		
**Waist-hip ratio**			
Height	0.21	0.03	< 0.001
Marital status			
Single	0.08	0.01	< 0.001
Widowed	0.04	0.004	< 0.001
Divorced	0.02	0.004	< 0.001
Married/unmarried with a partner	0^a^		
Negative life events			
Yes	0.01	0.004	0.033
No	0^a^		

Note: analyzed by by generalized estimating equations

## Discussion

This study sheds light on worsening trends in the nutritional status of perimenopausal women using the first large-sample multi-centered longitudinal study in Southwest China. By analyzing the specific body composition variables in addition to BMI and weight, we determined a more comprehensive profile of the nutritional status in perimenopausal women: average weight was found to keep increasing among perimenopausal women over time, accompanied by increased fat mass and waist-hip ratio, and decreased protein level, skeletal muscle, and total body water. By exploring the influences of sociodemographic variables on nutritional status, these results may assist in the screening of key populations which are at risk of malnutrition in this critical life stage. Our findings are important and relevant to health professionals in both clinic and community settings and can be used to guide the formulation of precision nutrition policies and intervention measures.

At our baseline assessment (T1), 20.1% of participants who were overweight (17.4%) and obese (2.7%). Researchers from the United States reported that two-thirds of middle-aged women were overweight [14], which is much higher than our findings. Although the prevalence of overweight and obese women is much lower in our study, what is of interest is that participants’ weight increased by 1 kg on average over one year. This value is nearly twice that reported in the United States (+ 0.68 kg) [13]. Different study populations and body types might have contributed to these differences in findings. In addition, the faster accumulation of weight in our population may be associated with the characteristics of Sichuan food [19] and the famed casual lifestyle. In addition to weight changes, we also investigated the longitudinal trend of BMI in our sample, finding no significant variation, which is in line with previous findings [17, 23]. Given the rate of weight accumulation in our sample, longer follow-up durations are needed to fully understand the trajectories in both weight and BMI.

Our study adds information to the existing literature by exploring the changes in specific body composition in addition to weight and BMI. According to our findings, weight gain during perimenopause is dominated by an increase in fat mass accompanied by a decrease in other important body composition indicators, including skeletal muscle, total body water, and protein. These findings are in line with published literature [17, 24, 25]. For example, a longitudinal study involving 1246 Americans of different races reported that accelerated gains in fat mass and losses of lean mass are perimenopause-related phenomena [17]. The probable mechanisms underlying these changes are mainly related to metabolic syndrome regulated by dysfunctional ovaries during perimenopause [26]. Concretely, insulin resistance, abdominal obesity, and dyslipidemia dominate metabolic syndrome during the menopause transition [27], hence, the continuous increase in fat mass is not surprising. Insulin resistance [28] has been proven to be a risk factor for muscle catabolism, and water accounts for approximately 75% of the muscle mass [29]. This is evidenced in our findings that total body water decreased along with the diminishment in skeletal muscle. Moreover, other researchers hypothesize [8] that skeletal muscles reduce their capacity for activated protein synthesis in response to anabolic stimuli induced by insulin resistance, which, combined with the increased protein requirements associated with the aging process [30] is a good illustration of the decreasing protein trend in our findings.

Since the nutritional indicators were all measured in units of mass, it is not surprising that higher heights would therefore result in higher values for all the variables adopted in our study. We also found that older age, lower educational level, lower income, and negative life events are associated with nutrition risk. A previous study also documented the connection between age and nutritional status risk [31]. Conversely, with an increase in education and income, individuals tend to adopt a healthier lifestyle [32], which may contribute to better nutritional outcomes. However, marital status and negative life events undermine individuals’ nutritional status by harming their social relations and positive outcomes such as life satisfaction and quality of life [33]. Therefore, our findings suggest that perimenopausal women of different socioeconomic backgrounds have different risks for malnutrition, and precision intervention should be considered based on the screening of the most vulnerable population.

Our study has several limitations. Firstly, in addition to the sociodemographics included in our study, nutritional status is influenced by other factors such as dietary consumption, physical activity, sleep, and some specific diseases. These factors should be included in future studies to expand and support our findings. Secondly, a potential educational intervention on dietary habits during the longitudinal survey was unavoidable, and its potential effects on participants’ behavior should also be documented in the future. Participants from other regions of China should be recruited, and randomization should be adopted to increase the generalization of our findings. Thirdly, the duration of perimenopause varies from person to person, and it can last from less than one year to more than ten years [34]; studies with a longer follow-up should be undertaken. Fourthly, we did not measure anthropometric and body compositions at the same time of day for all visits. This might result in measurement deviation and should be considered in the future to further increase the accuracy of our findings. Lastly, our study was carried out from 2021 to 2022 and the influences of the COVID-19 pandemic could not be excluded, therefore future interventional studies are needed to further strengthen our findings.

## Conclusions

There are worsening trends in perimenopausal women’s nutritional status. Weight continued to increase, dominated by the accumulation of fat mass and the diminution of other nutritional indicators, including skeletal muscle, total body water, and protein. Perimenopausal women with older age, lower income, less education, being unmarried and with negative life events are especially at risk of malnutrition. Precision nutritional interventions are needed, and recipients should be classified into different risk levels based on their sociodemographic background for greater efficiency in care. Our findings need to be strengthened by future studies.

## Data Availability

The datasets used and/or analyzed during the current study will be available from the corresponding author on reasonable request.
